# Supplementary material for: Effective connectivity in a duration selective cortico-cerebellar network
Source: Sci Rep. 2023 Nov 24;13:20674. doi: 10.1038/s41598-023-47954-4 (PMC10673930; doi:10.1038/s41598-023-47954-4)
Supplement: Supplementary file 1 — Supplementary Information. [file 41598_2023_47954_MOESM1_ESM.docx]

**Supplementary Material**

**Effective Connectivity in a Duration Selective Cortico-Cerebellar Network**

Foteini Protopapa, Shrikanth Kulashekhar, Masamichi J. Hayashi, Ryota Kanai and Domenica Bueti


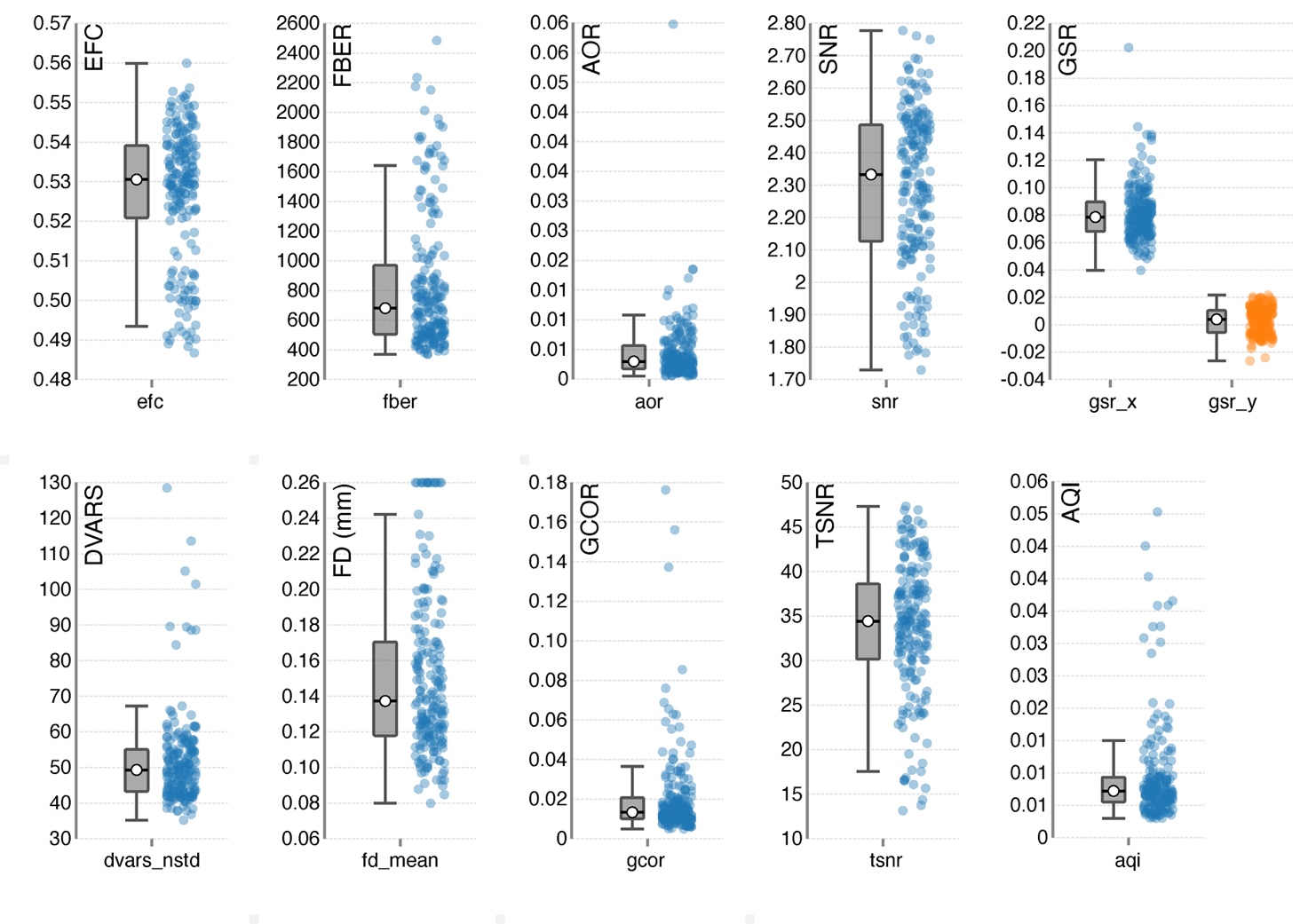


**Supplementary Figure 1 Image Quality Control.** The quality control measures were estimated individually for each subject and each fMRI run using MRIQC tool. The figure shows the average value of the metric for each of the subject and run. The boxplots shown are Tukey boxplots, with median and interquartile range. Whiskers extend to the farthest point that is within 1.5 * IQR of the upper (or lower) quartile. Entropy-Focus Criterion (EFC) indicates blurring, and ghosting caused by head motion. Foreground-Background Energy Ratio (FBER) is the mean energy of image values within the head, relative to the energy of the image values in the air mask. AFNI’s outlier ration (AOR) represents the mean fraction of outliers in each volume. Static Signal to Noise Ratio (SNR) is the mean intensity within the gray matter divided by the standard deviation of the values outside the brain. Ghost to signal ratio (GSR) is the ratio between the mean signal intensity in areas with apparent ghosting and the signal intensity of the brain.  DVARS (D: Temporal Derivative of Time Courses, Vars is the Root Mean Square (RMS) Variance Over Voxels) shows the rate of change of BOLD signal across the entire brain at each timepoint. Framewise Displacement (FD) is defined as the sum of the absolute values of six realignment parameters at each timepoint, after converting rotational displacements from degrees to millimeters. Global correlation (GCOR) is the average correlation between every voxel and every other voxel over a given mask. Temporal signal to noise ratio (tSNR) is the ratio between mean signal of time series and the temporal standard deviation. AFNI’s quality index (AQI) is the mean distance between each volume and the median volume.

**
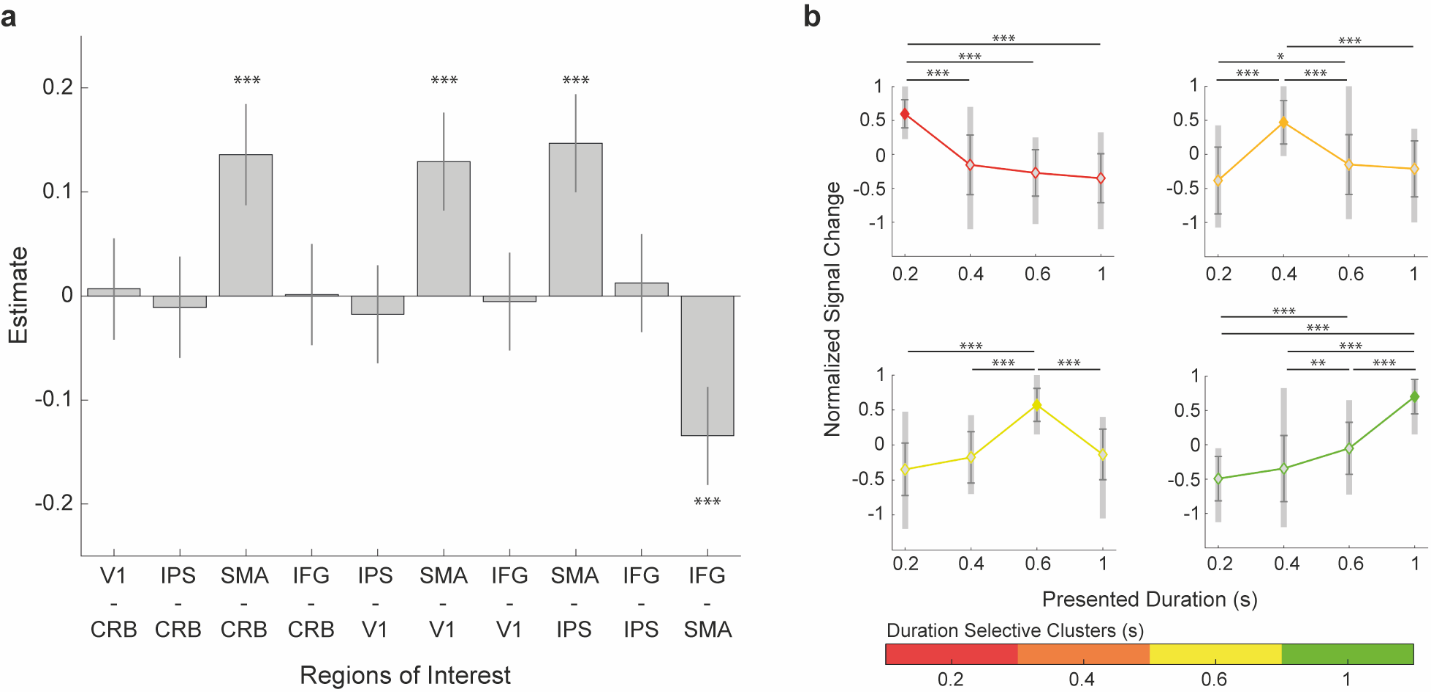
**

**Supplementary Figure 2 Cross-validated tuning. (**a) Plot of the differences between estimated BOLD response (y axis) in the different ROIs (x axis). (b) Plot of the estimated BOLD response averaged across all ROIs (y axis) in different duration selective clusters of voxels (colored lines in different subplots) in response to different presented durations (x axis). The shaded grey area in the plots is the distribution across the 18 iterations of splits (i.e., 9 runs to select the duration selective clusters of voxels and 9 to extract BOLD), black bars are standard errors.

**
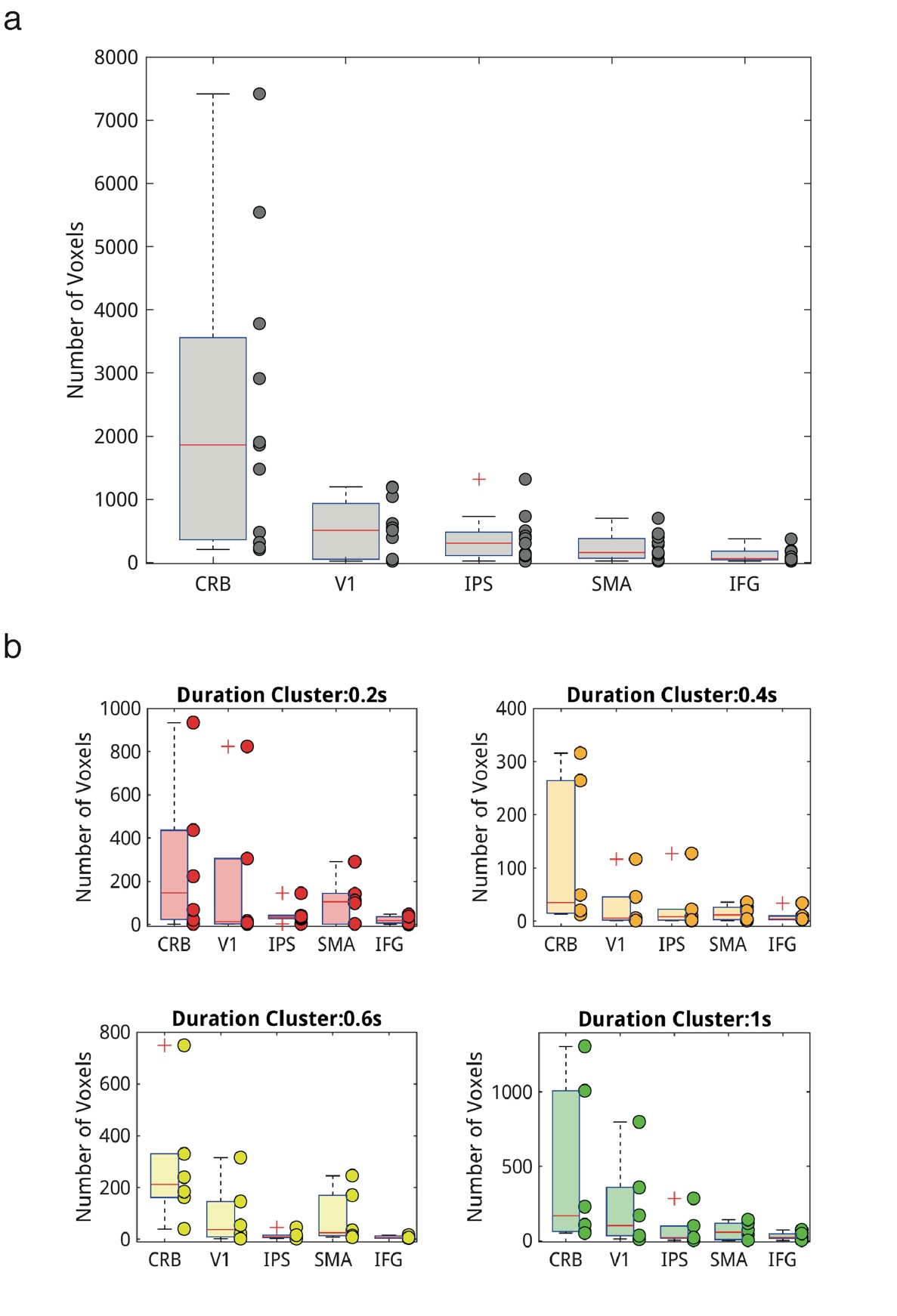
**

**Supplementary Figure 3. Number of voxels used in the 5-nodes and 20-nodes DCM analyses.**

In the boxplot, the central red mark indicates the median, and the bottom and top edges of the box indicate the 25th and 75th percentiles, respectively. The whiskers represent the most extreme data points without the outliers, while the '+' symbol represents the outlier. (a) The voxel sizes of the 5 ROIS (mean ± standard error): CRB (2375.8±714.0), V1 (513.5±139.3), IPS (374.5±114.6), SMA (245.0±63.6) and IFG (115.5±32.5). (b) The voxel sizes of the durations clusters for the 5 ROIs. The voxel sizes for the clusters of the duration 0.2s: CRB (280.8±108.1), VI (193.0±99.9), IPS (47.2±14.9), SMA (108.0±32.2) and IFG (21.0±5.4). The sizes for the clusters of the duration 0.4:  CRB (113.2±41.8), V1 (29.7±13.9), IPS (28.5±14.7), SMA (15.0±4.4) and IFG (9.8±3.7). The number of voxels for the clusters of the duration 0.6s: CRB (284.7±74.5), V1 (91.7±36.7), IPS (15.7±4.7), SMA (82.0±30.4) and IFG (9.0±1.6).The voxel sizes for the clusters of the duration 1s: CRB (459.5±166.3), V1 (233.7±92.4), IPS (72.8±32.7),  SMA (63.2±17.3) IFG (29.0±7.7).


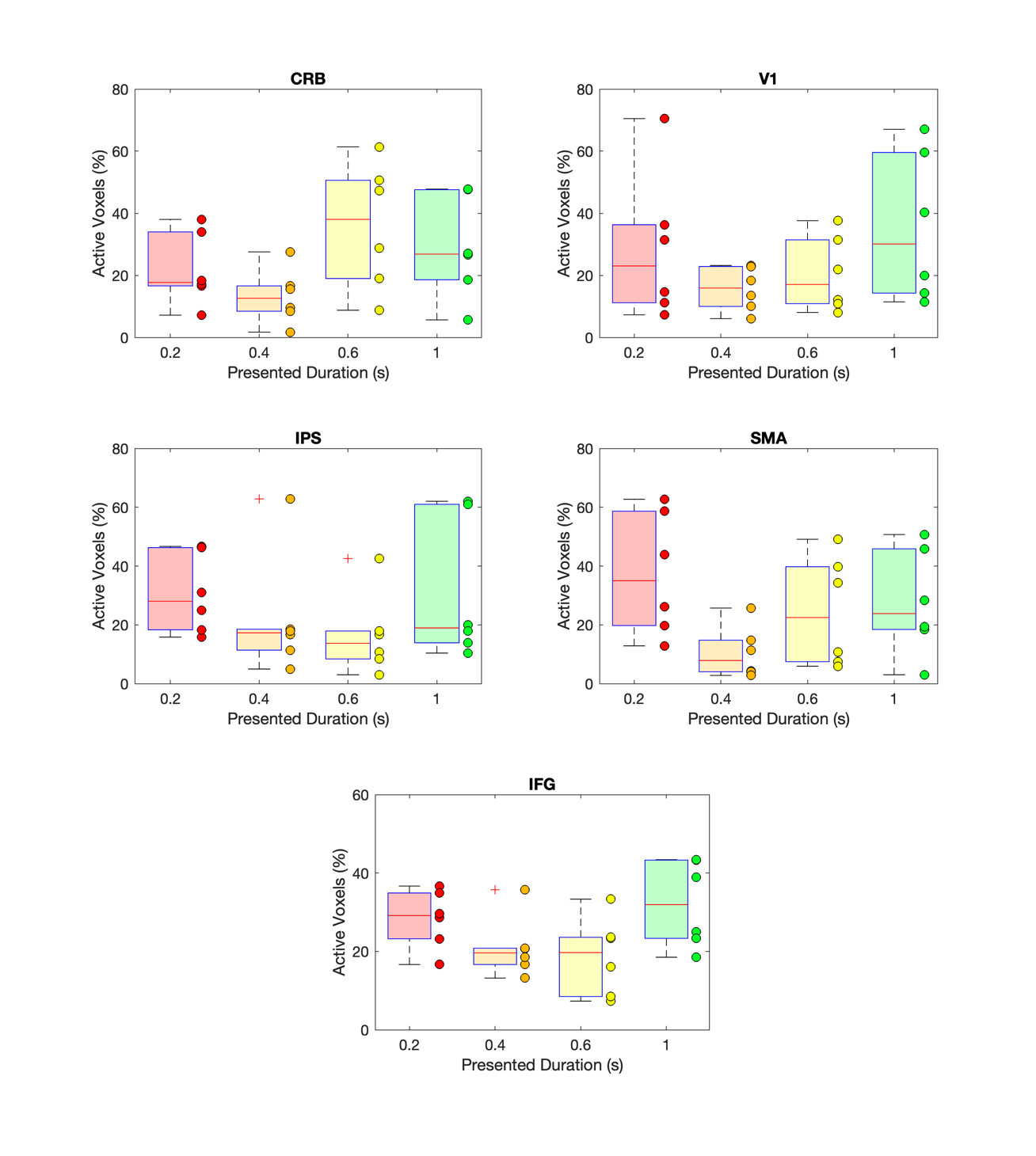


**Supplementary Figure 4 Proportion of duration selective voxels in each ROI**

For each ROI, the proportion of active duration selective voxels (number of active voxels/ total number of active voxels within the ROI) is plotted. These are the clusters of voxels used in 20-nodes DCM analyses (N subjects =6). Bars are color coded according to the duration preference. In the boxplot, the central red mark indicates the median, and the bottom and top edges of the box indicate the 25th and 75th percentiles, respectively. The whiskers represent the most extreme data points without the outliers, while the '+' symbol represents the outlier.


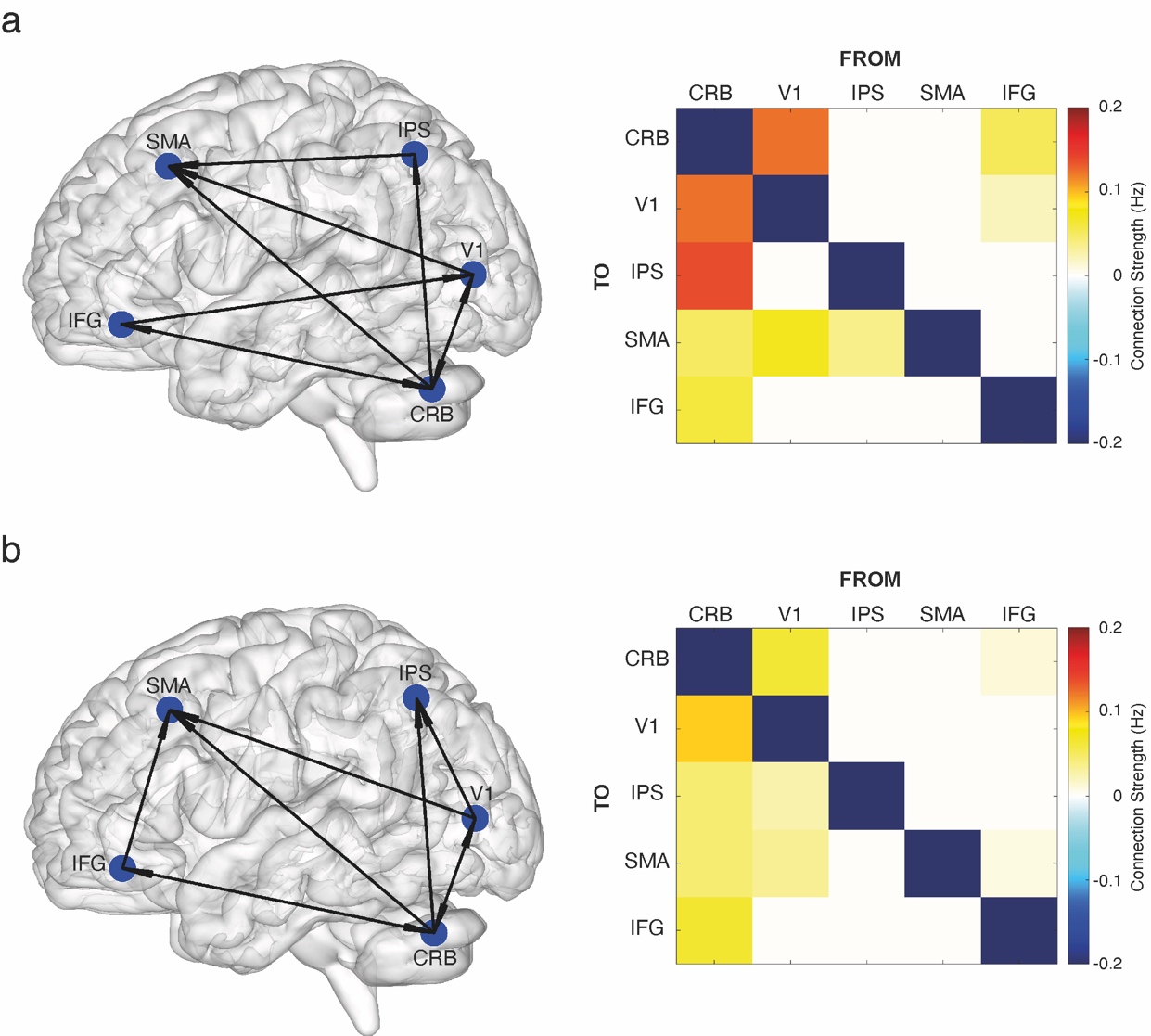


**Supplementary Figure 5.**  On the left-hand side is a graph representation of the network architecture (A-matrix) as resulted from the 5-nodes cross-validated PEB analysis. The directionality of connections is depicted with arrows. On the right-hand side is the matrix of the strength of the survived connections after the pruning of the parameters done by PEB. Values are in Hz (see colorbar). In the cross-validated PEB for each subject we used 9 fMRI runs to define the ROIs and the other 9 to run the PEB. Legend: CRB= cerebellum, V1=primary visual cortex, IPS= intraparietal sulcus, SMA=supplementary motor area, IFG= inferior frontal gyrus.

**
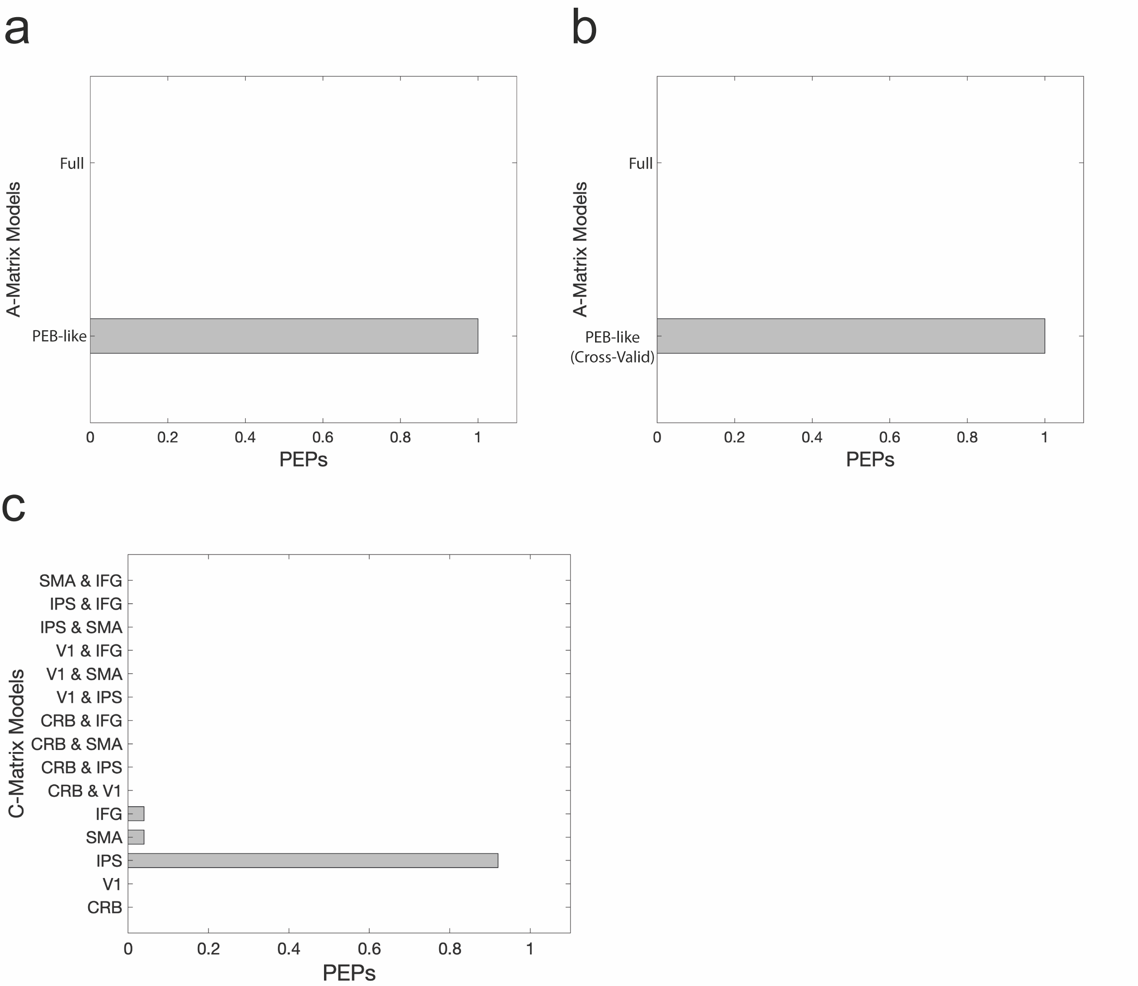
**

**Supplementary Figure 6. Bayesian model selection (BMS) and Bayesian model comparison results.**

(a, b) Protected exceedance probabilities (PEPs) computed to compare two models with distinct connectivity structures (A-matrix): a “fully connected” and “PEB-like”. In (a) is the original PEB-like structure in (b) is the cross-validated PEB version of it. In both models, B and C matrices were left fully modulated. We considered mandatory for a “winning” model to have PEPs greater than 90%. (c) PEPs of the 15 models we compared to find out which region(s) among the five ROIs was modulated by stimulus presentation (C-matrix). Fifteen possible models with 15 different combinations of C-matrices were tested. In the C-matrices of these 15 models, we arbitrarily allowed to have one or maximum two brain regions modulated by event duration. All models had an A-matrix “PEB-like” and a B-matrix in which all existing connections were modulated by stimulus presentation.

**
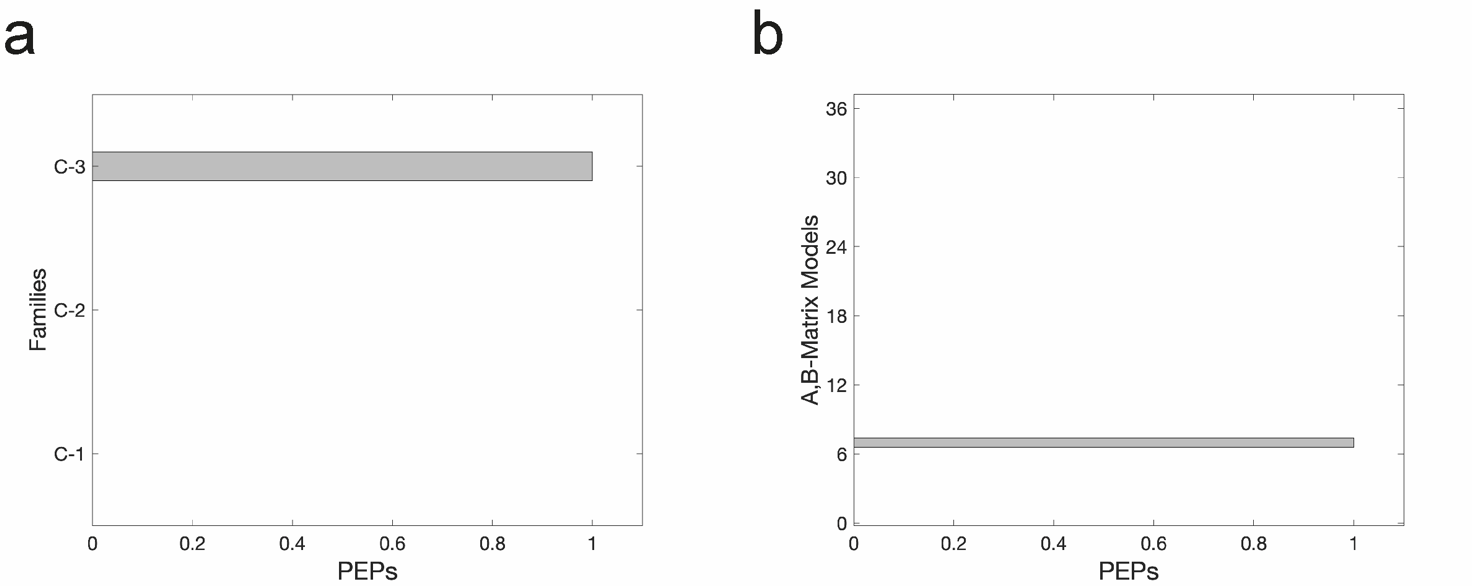
**

**Supplementary Figure 7. Bayesian model selection (BMS) and Bayesian model comparison results.**

a) BMS among three families of models that differ according to the modulatory effect of stimulus duration on the activity of IPS duration selective clusters (i.e., C-matrix that could be: stimulus *duration independent*, *neighboring dependent,* or stimulus *duration dependent*). Bar plot shows their protected exceedance probabilities (PEPs). Each family consists of 36 models (108 models in total). The winning family is the family of models where the modulation by S1 stimulus duration on IPS activity is stimulus *duration dependent*.

(b) PEPs of the 36 models of the winning family as shown in panel a (activity of IPS modulated in a duration dependent fashion), that differ in connectivity (A-matrix) and modulation of connectivity by stimulus duration (B-matrix) according to the different levels of duration specificity (i.e., stimulus *duration independent*, *neighboring dependent,* or stimulus *duration dependent).* The winning model is the model with an A-matrix “PEB-like” duration independent (i.e., fully-connected) and a B matrix *duration dependent*.


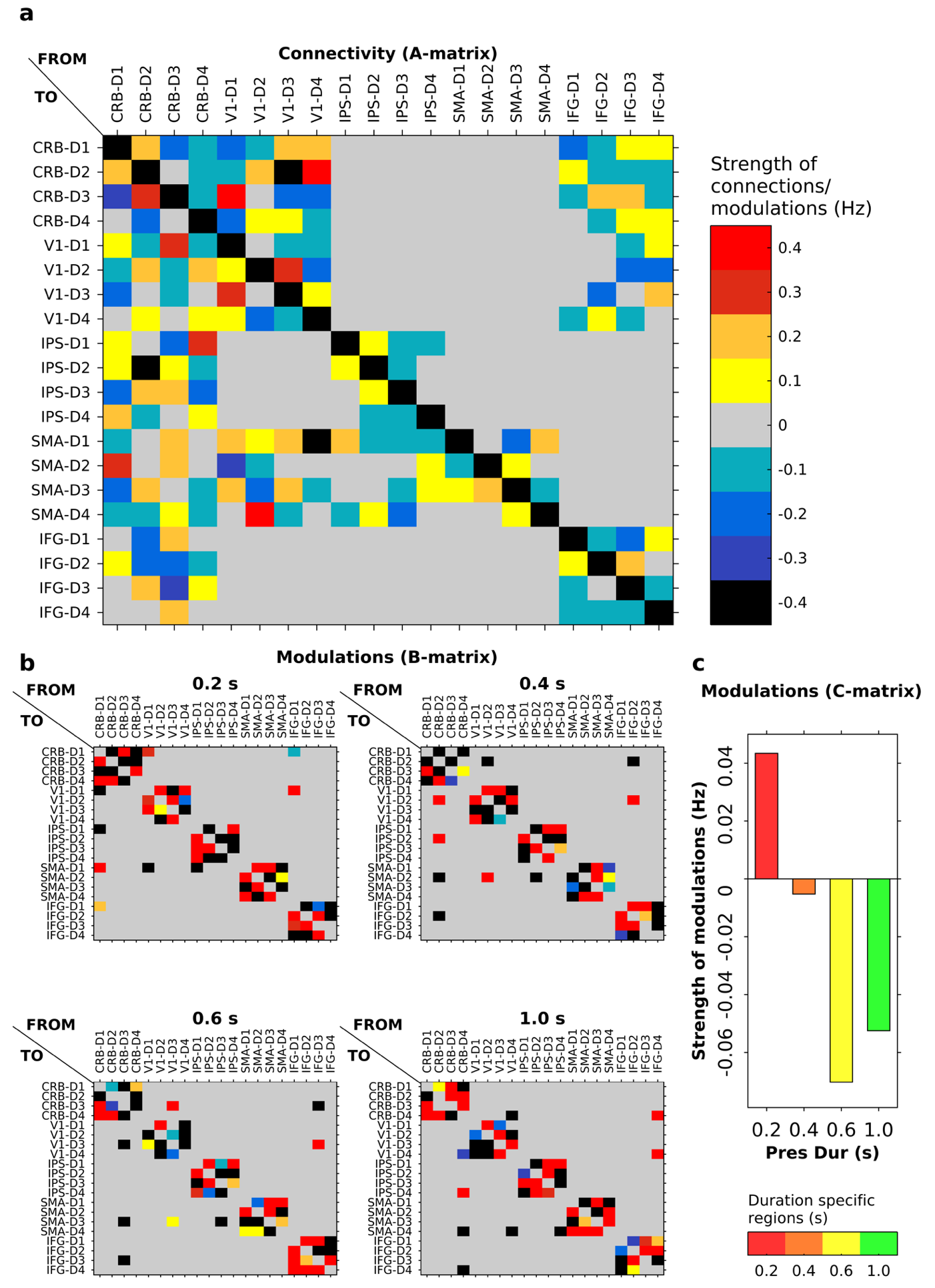


**Supplementary Figure 8 Bayesian model averaging (BMA) results of the winning model.** BMA averages the A-B-C-parameters of the winning model across subjects and sessions. (a) The matrix represents the strength of connections (A-matrix) between duration selective clusters of voxels. The color scale represents the parameter’s values. These values range from -0.4 to 0.4 Hz. (b) The four matrices represent the modulation on the connectivity strength (B-matrix) between the different duration selective clusters at the offset of the four S1 durations. x and y-axes are as in (a). (c) Bar-plot of the activity within each IPS duration selective cluster (C-matrix). Each bar shows the neuronal change (y-axis) for each of the four durations specific clusters while a specific duration was presented (x-axis). Duration specific clusters and stimulus durations are color coded as follow: red= 0.2, orange=0.4, yellow=0.6, and green= 1 s.

**
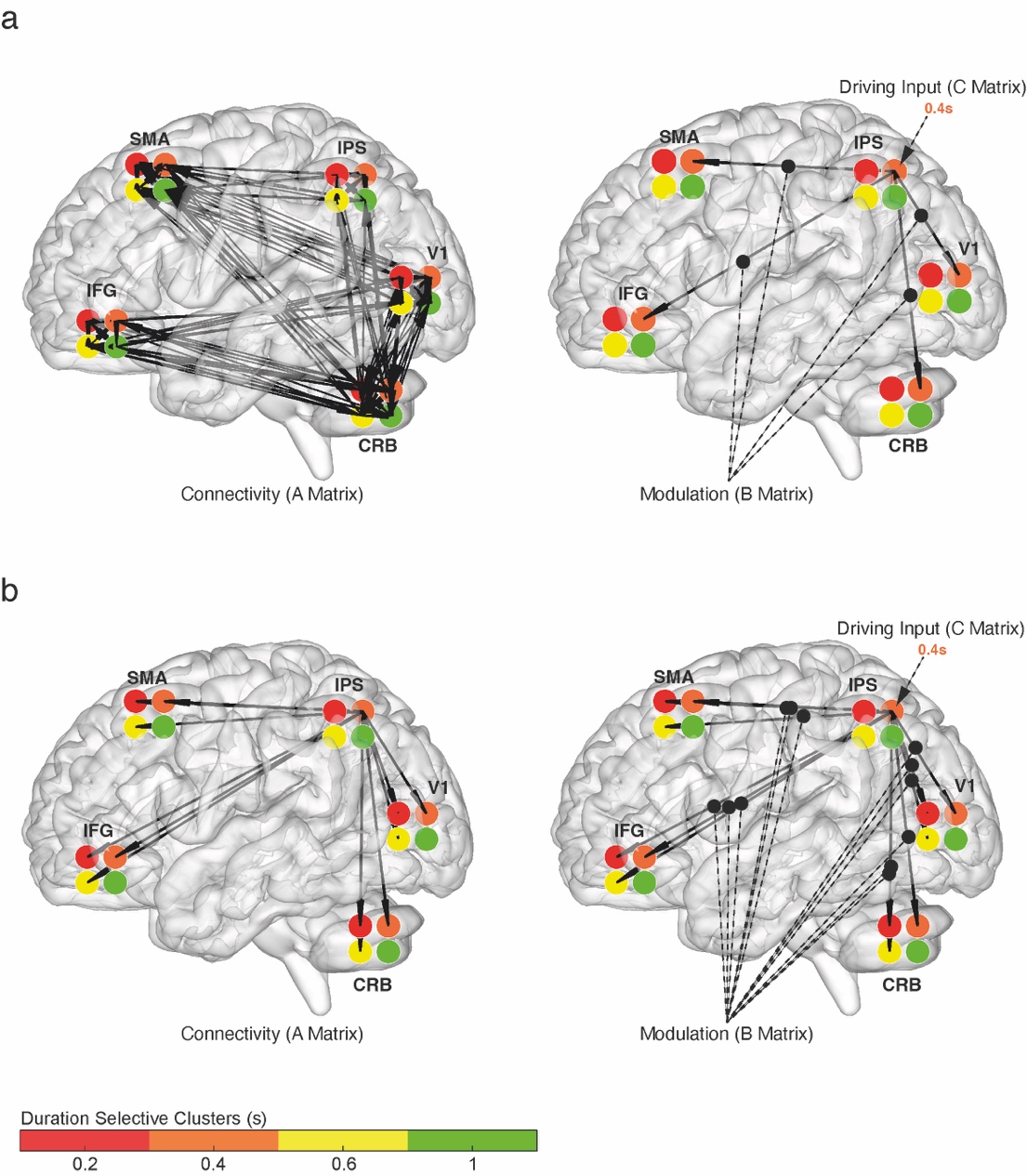
**

**Supplementary Figure 9** **Winning cross-validated model of the DCM 20-nodes network.** Graph representation of the winning model conducted on half of the dataset (9 runs were used to define the ROIs and on the other 9 we ran the DCM). On the left-hand side is the A-matrix, on the right-hand side the B and C matrices. The network architecture and the modulatory effect of stimulus presentation on connectivity strength are both neighboring dependent while the driving input in IPS is duration dependent.


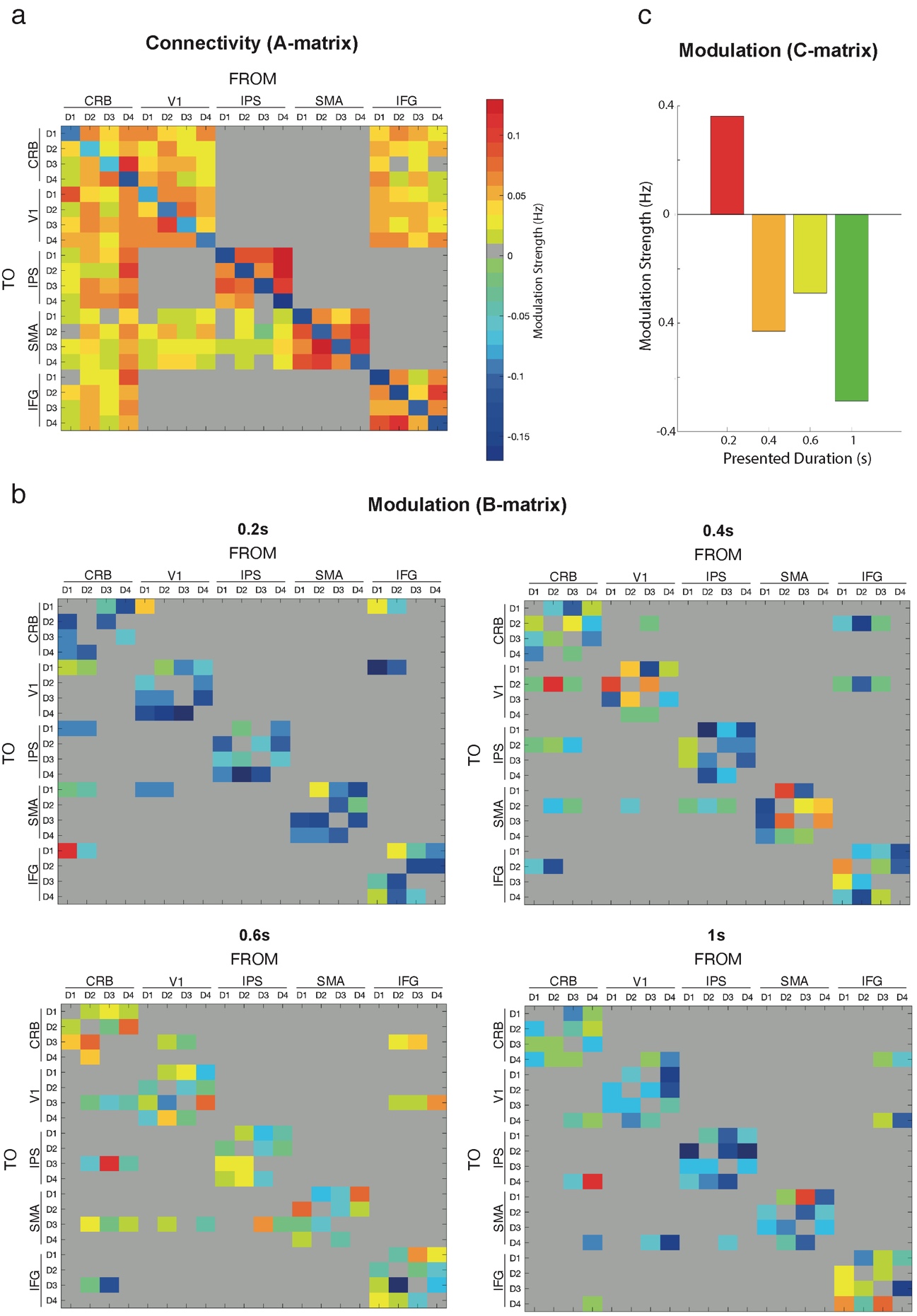


**Supplementary Figure 10 Bayesian model averaging (BMA) results of the winning model in the cross-validated 20-nodes DCM.** BMA averages the A-B-C-parameters of the winning model across subjects and sessions. (a) The matrix represents the strength of the connections (A-matrix) between duration selective clusters of voxels. The color scale represents the parameter’s values. (b) The four matrices represent the modulation on the connectivity strength (B-matrix) between the different duration selective clusters at the offset of the four S1 durations. x and y-axes are as in (a). (c) Bar-plot of the activity within each IPS duration selective cluster (C-matrix). Each bar shows the neuronal change (y-axis) for each of the four durations specific clusters while a specific duration was presented (x-axis). Duration specific clusters and stimulus durations are color coded as follow: red= 0.2, orange=0.4, yellow=0.6, and green= 1 s.


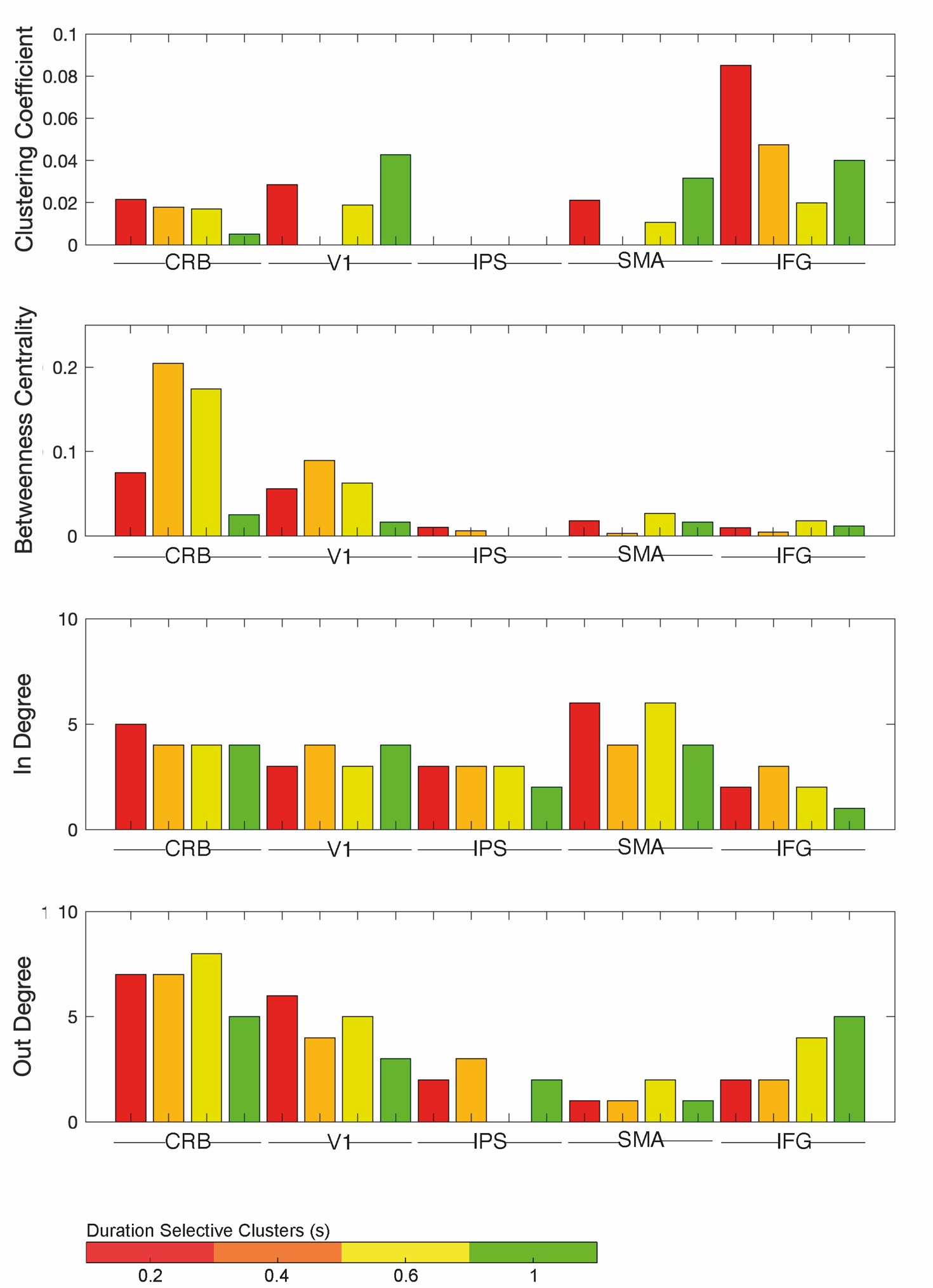


**Supplementary Figure 11.** **Graph analysis output.**  Graph metrics from the estimated effective connectivity A matrix of the 20-nodes DCM. The *clustering coefficient* is a measure of the presence of clusters in a graph. B*etweenness centrality* indexes the influence of a node over the flow of information in a graph. *In-degree* is a measure of the number of connections that arrive at a given node, whereas *out-degree* is the number of connections that leaves the node. The *x-axis*refers to the four duration selective clusters of the five ROIs.

| **Anatomical Regions** | **DARTEL coordinates (mm)** | | | **Cluster size** | **p_FWE_** | **T** |
| --- | --- | --- | --- | --- | --- | --- |
| Left Inferior Parietal Lobule | -40 | -40 | 40 | 2524 | < 0.001 | 7.93 |
| Left Supplementary Motor Area | -2 | 9 | 46 | 699 | < 0.001 | 7.50 |
| Right Mid-Occipital Gyrus | 21 | -98 | 12 | 152 | 0.002 | 6.63 |
| Right Lateral Cerebellum | 32 | -51 | -20 | 433 | < 0.001 | 6.43 |
| Left Inferior Frontal Gyrus | -50 | 4 | 10 | 736 | < 0.001 | 6.42 |
| Right Cerebellar Vermis | 9 | -72 | -14 | 305 | < 0.001 | 5.89 |
| Right Lingual Gyrus | 18 | -45 | 2 | 264 | < 0.001 | 5.86 |
| Right Frontal Eye Fields | 27 | -6 | 52 | 122 | 0.009 | 5.59 |
| Left Calcarine Sulcus | -14 | -64 | 15 | 242 | < 0.001 | 5.42 |
| Right Middle Occipital Gyrus | 48 | -69 | 8 | 125 | 0.008 | 5.13 |
| Right Intraparietal Sulcus | 44 | -36 | 50 | 156 | 0.002 | 5.10 |
| Left Frontal Eye Fields | -24 | -6 | 46 | 219 | < 0.001 | 4.98 |

**Supplementary Table 1**

Stereotaxic Dartel-11 coordinates (mm) for brain areas activated at the offset of the four S1 durations. Voxels activated at p<0.05, FWE cluster-level corrected for multiple comparisons across the whole brain volume.

| **Networks** | **Analysis** | **A-matrix** | **B-matrix** | **C-matrix** | **N models** |
| --- | --- | --- | --- | --- | --- |
| **5-nodes** | PEB | all | all | all | - |
|  | DCM | all | all | all | 1 |
|  | DCM | PEB-like | all | all | 1 |
|  | DCM | PEB-like | all | 1 or 2 ROIs | 15 |
| **20-nodes** | DCM | PEB-like  I,N,D | I,N,D | I,N,D | 108 |
|  | DCM | PEB-like  I,N,D | I,N,D | D | 36 |

**Supplementary Table 2** Summary of the analyses performed.

Legend: PEB = Parametric Empirical Bayesian, DCM=Dynamic Causal Modelling, I=*duration independent*, N=*neighboring* *dependent*, D=*duration* *dependent*
